# Reconstruction of combined bone and soft tissue defects of the hand and foot with free superficial circumflex iliac artery perforator osteocutaneous flaps: a retrospective analysis

**DOI:** 10.3389/fsurg.2026.1748242

**Published:** 2026-03-13

**Authors:** Jiadong Pan, Chenxi Zhang, Gaoxiang Yu, Luzhe Wu, Shanqing Yin, Xin Wang

**Affiliations:** 1 Department of Hand Microsurgery and Plastic Reconstructive Surgery, Ningbo No.6 Hospital, Ningbo, Zhejiang, China; 2 Ningbo Clinical Research Center for Orthopedics, Sports Medicine & Rehabilitation, Ningbo, Zhejiang, China

**Keywords:** bone flap, combined tissue defect, functional reconstruction, surgical flaps, vascularized bone graft

## Abstract

**Objective:**

To explore the surgical method and clinical efficacy of free superficial circumflex iliac artery perforator osteocutaneous flap in repairing combined bone and soft tissue defects of the hand and foot.

**Methods:**

From October 2011 to June 2023, 62 patients with combined bone and soft tissue defects of the hand and foot were treated with free superficial circumflex iliac artery perforator osteocutaneous flap, including 16 cases in the hand and 46 cases in the foot. The causes of injury included 18 cases of machine crush injury, 7 cases of traffic accident injury, and 37 cases of donor foot lesions after thumb/finger reconstruction. The area of skin defects ranged from 4 cm × 2 cm to 13 cm × 5 cm, and the length of bone and/or joint defects was 2 cm–8 cm. The flap harvesting range was 4.5 cm × 3.0 cm to 14 cm × 5.5 cm, and the volume of the iliac bone flap was 2 cm × 1 cm × 1 cm to 8 cm × 2.0 cm × 1.5 cm.

**Results:**

Postoperatively, 58 flaps survived uneventfully, and 4 cases developed vascular crisis, all of which survived after symptomatic treatment. The postoperative follow-up period was 10–59 months. The appearance and texture of the flap were good in 49 cases, and 13 cases underwent secondary revision surgery due to flap bulkiness. Bone union time was ≤3 months in 46 cases, 3–6 months in 12 cases, and more than 6 months in 4 cases. The last x-ray examination showed that 56 cases had complete morphology of the iliac bone flap, 3 cases had bone resorption, 1 case had non-union, and 2 cases had secondary fractures. The scar at the hip donor site was well-concealed, without obvious deformity or pain.

**Conclusion:**

The superficial circumflex iliac artery perforator osteocutaneous flap is an effective method for repairing combined bone and soft tissue defects of the hand and foot, with significant advantages at the donor site, while also posing certain technical challenges.

## 1 Introduction

The repair of combined bone and soft tissue defects in the hand and foot poses significant challenges, as the duration, methods, and outcomes of treatment are closely correlated with the recovery of the appearance and functional rehabilitation of the patient's hand and foot (1). The protocol of primary flap coverage for wound closure combined with bone cement packing for bone defects, followed by secondary bone grafting within the induced membrane, can effectively address the challenges of soft tissue repair and bone defect reconstruction, respectively. However, it is associated with drawbacks such as multiple surgical procedures and prolonged treatment and rehabilitation periods. In contrast, performing flap surgery and bone grafting simultaneously avoids these disadvantages but carries risks including bone resorption, deep infection, delayed union, and even nonunion. Therefore, free osteocutaneous flap transplantation represents a viable option for achieving faster and better reconstruction of such combined defects.

Currently, commonly used osteocutaneous flaps include the fibular, iliac, scapular, and lateral arm osteocutaneous flaps. For most bone defects in the hand and foot, the ilioabdominal region serves as an ideal donor site. In 1979, Taylor (2) first described the iliac osteocutaneous flap nourished by the deep circumflex iliac artery (DCIA), noting that a DCIA-based pedicle provides abundant blood supply to the iliac bone and a sufficient volume of cancellous bone. Nevertheless, the deep anatomical location of the DCIA and its complex local anatomy result in longer operative times, increased technical difficulty, and potential postoperative complications such as lateral thigh numbness and abdominal wall herniation.

To overcome these limitations, building upon the superficial circumflex iliac artery (SCIA) perforator flap first described by Koshima (3) in 2004, we designed the SCIA perforator osteocutaneous flap utilizing the perforating vessels from the deep branch of the SCIA that supply the anterior iliac crest. This flap has been used for simultaneous reconstruction of combined bone and soft tissue defects in the hand and foot, yielding favorable outcomes. In this study, we retrospectively analyzed 62 cases treated at our center over a decade to summarize the technical essentials of the SCIA perforator osteocutaneous flap. Clinical efficacy was evaluated using primary outcomes such as clinical bone union time and bone resorption, along with secondary outcomes including donor and recipient site appearance and functional recovery. The collection of case data in this study was approved by the Medical Ethics Committee of Ningbo Sixth Hospital (2022-12K).

## 2 Method

### 2.1 Selection criteria and indicative profiles

#### 2.1.1 Inclusion criteria

(1) Traumatic hand/foot composite defects. (2) donor foot lesions after digit reconstruction. (3) Primary repair with free SCIA flaps. (4) Negative preoperative wound cultures; minimum 12-month follow-up.

#### 2.1.2 Exclusion criteria

(1) Staged reconstruction; severe systemic microangiopathy (e.g., Buerger's disease). (2) active chronic osteomyelitis; cardiopulmonary instability.

#### 2.1.3 Indications

(1) Need for thin skin; bone loss of 2–8 cm. (2) Desire to preserve major limb vessels (peroneal or radial arteries).

#### 2.1.4 Comorbidities

①Patients with controlled hypertension (*n* = 15). ②type 2 diabetes (*n* = 8), and hyperlipidemia (*n* = 5) were included following medical optimization.

### 2.2 General data

There were 47 males and 15 females in this group, aged 17–64 years with a mean age of 38.6 years. Etiologies included 18 cases of machine crush injuries, 7 cases of traffic injuries, and 37 cases of donor foot lesions following thumb/finger reconstruction. Recipient sites comprised 16 cases in the hand (7 on the left, 9 on the right) and 46 cases in the foot (15 on the left, 31 on the right). Comorbidities included 15 cases of hypertension, 8 cases of type 2 diabetes mellitus, and 5 cases of hyperlipidemia.

All traumatic cases initially underwent wound debridement and negative pressure drainage. Tissue reconstruction surgery was performed only after no signs of infection were observed at the defect sites. Lesions in the donor foot after thumb/finger reconstruction resulted from harvesting of the hallux toenail flap, hallux toenail flap with a common vascular pedicle, or second toe bone-tendon flap, all of which were repaired concurrently using free superficial circumflex iliac artery perforator osteocutaneous flap transplantation.

Skin defect areas ranged from 4 cm × 2 cm to 13 cm × 5 cm. Bone or/and joint defect lengths measured 2 cm–8 cm, involving 3 cases of phalanges, 7 cases of metacarpals, 6 cases of metacarpophalangeal bones, 28 cases of toe phalanges, 6 cases of metatarsals, 9 cases of metatarsophalangeal bones, and 3 cases of calcaneus. The harvested flap dimensions ranged from 4.5 cm × 3 cm to 14 cm × 5.5 cm, with bone flap volumes ranging from 2 cm × 1 cm × 1 cm to 8 cm × 2 cm × 1.5 cm (Table 1).

**Table 1:** Basic information of patients.

(Group)		Range and number	Mean/percentage	Median
Gender (*n*)	Male	47	75.80%	
	Female	15	24.20%	
Age (y)		17–64	38.6 ± 12.3	37.5
Affected Limbs (*n*)	Left hand	7	11.30%	
	Right hand	9	14.50%	
	Left foot	15	24.20%	
	Right foot	31	50.00%	
Cause of Injury (*n*)	Mechanical crush injury	18	29.00%	
	Traffic injury	7	11.30%	
	Lesion of donor foot after thumb/finger reconstruction surgery	37	59.70%	
Comorbidities (*n*)	Hypertension	15	24.20%	
	Type 2 diabetes mellitus	8	12.90%	
	Hyperlipidemia	5	8.10%	
Skin defect area (cm^2^)		4 × 2–13 × 5	32.4 ± 18.6	31.8
Bone/joint defect length (cm)		2–8	4.3 ± 1.8	4.5
Harvested skin flap area (cm^2^)		4.5 × 3–14 × 5.5	41.0 ± 21.2	39.3
Harvested bone flap volume (cm^3^)		2 × 1 × 1–8 × 2 × 1.5	7.9 ± 6.0	6.4

**Table 2:** Clinical outcomes and subgroup analysis (hand vs. Foot).

Variables	Total (*n* = 62)	Hand group (*n* = 16)	Foot group (*n* = 46)
Flap outcomes			
Flap survival, *n* (%)	58 (93.5%)	15 (93.8%)	43 (93.5%)
Vascular crisis, *n* (%)	4 (6.5%)	1 (6.3%)	3 (6.5%)
Secondary revision (Bulkiness), *n* (%)	13 (21.0%)	3 (18.8%)	10 (21.7%)
Bone healing outcomes			
Clinical bone union time (months)†	3.4 ± 1.5	3.2 ± 1.1	3.5 ± 1.6
Radiographic findings, *n* (%)			
Intact morphology	56 (90.3%)	15 (93.8%)	41 (89.1%)
Bone resorption	3 (4.8%)	1 (6.3%)	2 (4.3%)
Non-union	1 (1.6%)	0 (0.0%)	1 (2.2%)
Secondary fracture	2 (3.2%)	0 (0.0%)	2 (4.3%)
Donor site morbidity			
Wound dehiscence/infection	0 (0%)	0	0

### 2.3 Treatment methods

#### 2.3.1 Design of osteocutaneous flap

All operations were performed under general anesthesia or spinal anesthesia combined with brachial plexus block anesthesia. Preoperatively, color Doppler flow ultrasound was used to detect and mark the superficial branch, deep branch, and their main perforating vessels of the superficial circumflex iliac artery (SCIA). The flap was designed at the donor site according to the size and shape of the skin and soft tissue defect, following three principles: The axis of the flap was aligned as closely as possible with the course of the deep branch of the SCIA; The area of the flap was slightly larger than that of the wound; The anterior superior iliac spine (ASIS) was included within the flap. For defects with irregular shapes or excessive width, a lobulated flap was designed to ensure complete coverage of the wound after lobule splicing.

#### 2.3.2 Harvesting of osteocutaneous flap

First, an incision was made at the inferior margin of the flap. The flap was elevated between the superficial and deep layers of fat, and the superficial circumflex iliac vein (SCV) in the superficial layer was dissected and preserved for subsequent use. Combined with the preoperative localization results of SCIA perforators, the perforating vessels arising from the deep branch of the SCIA were carefully dissected under a head-mounted magnifying glass. After confirming that the perforators entered the skin, retrograde dissection of the perforators was continued in the deep adipose tissue until the deep branch of the SCIA was fully exposed.

Subsequently, dissection was performed along the deep branch toward the ASIS. After identifying the nutrient vessels supplying the ASIS (originating from the deep branch of the SCIA), the anterolateral femoral cutaneous nerve (CN)—which runs inferiorly along the anterior edge of the ASIS—was isolated and protected. The fascia surrounding the nutrient vessels of the ilium and between the flap and the iliac periosteum was retained to maximize the blood supply to the iliac bone graft.

**Figure 1:**
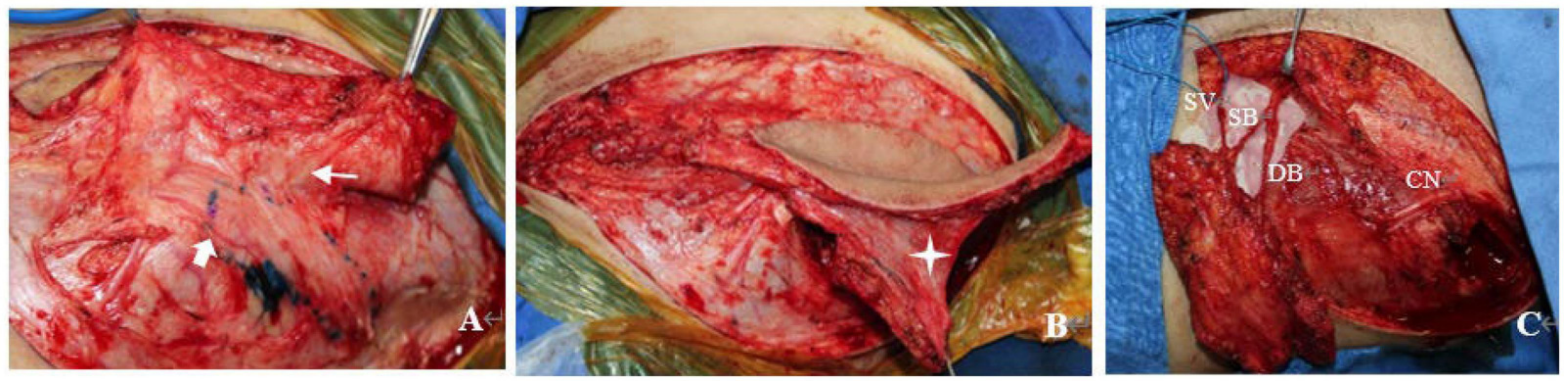
Harvesting of the perforating osteocutaneous flap of the superficial circumflex iliac artery (SCIA): **(A)** nutrient vessels originating from the deep branch of the SCIA are visible on the surface of the bone flap (thick arrow); meanwhile, a rich vascular network is observed in the fascia between the bone flap and the cutaneous flap (thin arrow). **(B)** On the premise of not affecting the placement of the iliac bone flap, a certain width of fascia between the iliac bone flap and the cutaneous flap is retained (four-pointed star), which helps to enhance the blood supply to the bone flap. **(C)** Appearance of the osteocutaneous flap before pedicle transection. Note the anatomical relationship among the superficial branch (SB) of the SCIA, deep branch (DB) of the SCIA, superficial circumflex iliac vein (SCV), and anterolateral femoral cutaneous nerve (CN).

Finally, an incision was made at the superior margin of the flap. The superficial and deep branches of the SCIA were dissected downward and proximally between the superficial and deep fat layers. According to the size of the bone defect, an iliac bone flap was harvested using a bone chisel slightly posterior to the ASIS. Bleeding from the osteotomy surface of the bone flap was observed in all cases (Figures 1A–C).

#### 2.3.3 Implantation of osteocutaneous flap and management of donor site

After pedicle transection of the osteocutaneous flap, the iliac bone graft was trimmed with rongeur forceps according to the requirements of the bone defect at the recipient site. The bone flap was then precisely inserted into the bone defect and fixed with Kirschner wires or miniature plate screws.

Vascular reconstruction for flap perfusion was performed via end-to-end anastomosis or end-to-side anastomosis, depending on the diameter of the blood vessels at the recipient site. At the bone harvest site, bone wax was used to achieve hemostasis. A drain was placed, and the donor site wound was closed by layered suturing.

### 2.4 Postoperative management

Patients were maintained on bed rest for 1 week postoperatively. Local warmth preservation was applied using a heat lamp. Anti-infective, antispasmodic, and anticoagulant therapies were administered routinely. After the osteocutaneous flap survived, patients were referred to the rehabilitation department for physical therapy and functional exercises under the guidance of rehabilitation physicians.

### 2.5 Postoperative follow-up

Patients were followed up regularly at 1 month, 3 months, 6 months, 9 months, and 1 year postoperatively via outpatient visits or WeChat. After 1 year, patients underwent irregular voluntary outpatient follow-ups. The follow-up content included: Appearance of the flap donor and recipient sites; Local pain symptoms; Healing status of the bone flap; Functional recovery of the hands and feet.

### 2.6 Statistical analysis

Statistical analysis was performed using SPSS version 26.0 software (IBM Corp., Armonk, NY, USA). Continuous variables, such as defect size, flap dimensions, and bone union time, were expressed as mean ± standard deviation (SD). Categorical variables, including the incidence of complications (e.g., vascular crisis, infection) and bone healing outcomes, were presented as frequencies and percentages (*n*, %).

## 3 Results

### 3.1 Flap survival and complications

Among the 62 patients, 58 flaps (93.5%) survived uneventfully. Vascular crises occurred in 4 cases (6.5%): three patients experienced venous congestion, which was successfully managed through suture removal and localized bloodletting; one patient developed an arterial crisis within 24 h postoperatively and was salvaged via surgical exploration and re-anastomosis. All flaps ultimately survived. During a mean follow-up of 25 months (range, 10–59 months), 49 patients (79.0%) reported satisfaction with the flap's contour and texture, while 13 patients required secondary deburring due to bulkiness.

### 3.2 Radiographic and clinical bone union

Bone union was assessed through serial x-ray or CT scans. Radiographic union was defined as the presence of continuous bridging trabeculae across the graft-host interface.

#### 3.2.1 Timeline

46 patients achieved union within 3 months, 12 within 3–6 months, 2 within 6–9 months, and 2 after 9 months.

#### 3.2.2 Outcomes

At the final follow-up, 56 patients (90.3%) demonstrated intact iliac bone morphology and solid incorporation.

#### 3.2.3 Complications

Bone resorption occurred in 3 cases and nonunion in 1 case. Two patients suffered secondary fractures. Two of these patients (one nonunion, one fracture) required secondary bone grafting to achieve final union. One patient with an asymptomatic intra-articular fracture at the foot donor site was managed conservatively with no functional deficit.

### 3.3 Functional recovery and donor site

Morbidity Functional outcomes were evaluated based on the restoration of limb integrity and mobility.

#### 3.3.1 Hand reconstruction

All patients regained protective sensation and demonstrated sufficient grip and pinch strength to return to their original occupations and perform activities of daily living independently.

#### 3.3.2 Foot reconstruction

Patients achieved stable, independent ambulation and full, painless weight-bearing. There were no reported impairments in wearing standard footwear, walking, or running.

#### 3.3.3 Donor site

At the iliac donor site, linear scars were aesthetically acceptable and inconspicuous. No patients reported chronic hip pain, gait instability, or sensory disturbances in the lateral femoral cutaneous nerve distribution.

### 3.4 Case 1

A 35-year-old male patient was admitted to hospital with an open fracture of the second metacarpal bone of the right hand (with bone defect) caused by mechanical crush injury. Clinical examination revealed:

A skin and soft tissue defect on the dorsal side of the right hand, measuring 5 cm × 2.5 cm; A bone defect length of 2.5 cm; Subluxation of the second metacarpophalangeal (MCP) joint; Contusion and necrosis of the intrinsic hand muscles on the radial side of the hand.

Emergency debridement, reduction of the dislocated joint and bone, and supportive internal fixation were performed to restore the alignment of the bone structural axis. Five days after the injury, the patient underwent reconstruction with a free perforator osteocutaneous flap of the superficial circumflex iliac artery (SCIA). The cutaneous component of the flap measured 6 cm × 3 cm, and the iliac bone flap had dimensions of 3 cm × 1 cm × 1 cm. The iliac bone flap was fixed with an internal plate; meanwhile, the collateral ligaments of the second MCP joint were repaired with the assistance of suture anchors.

**Figure 2:**
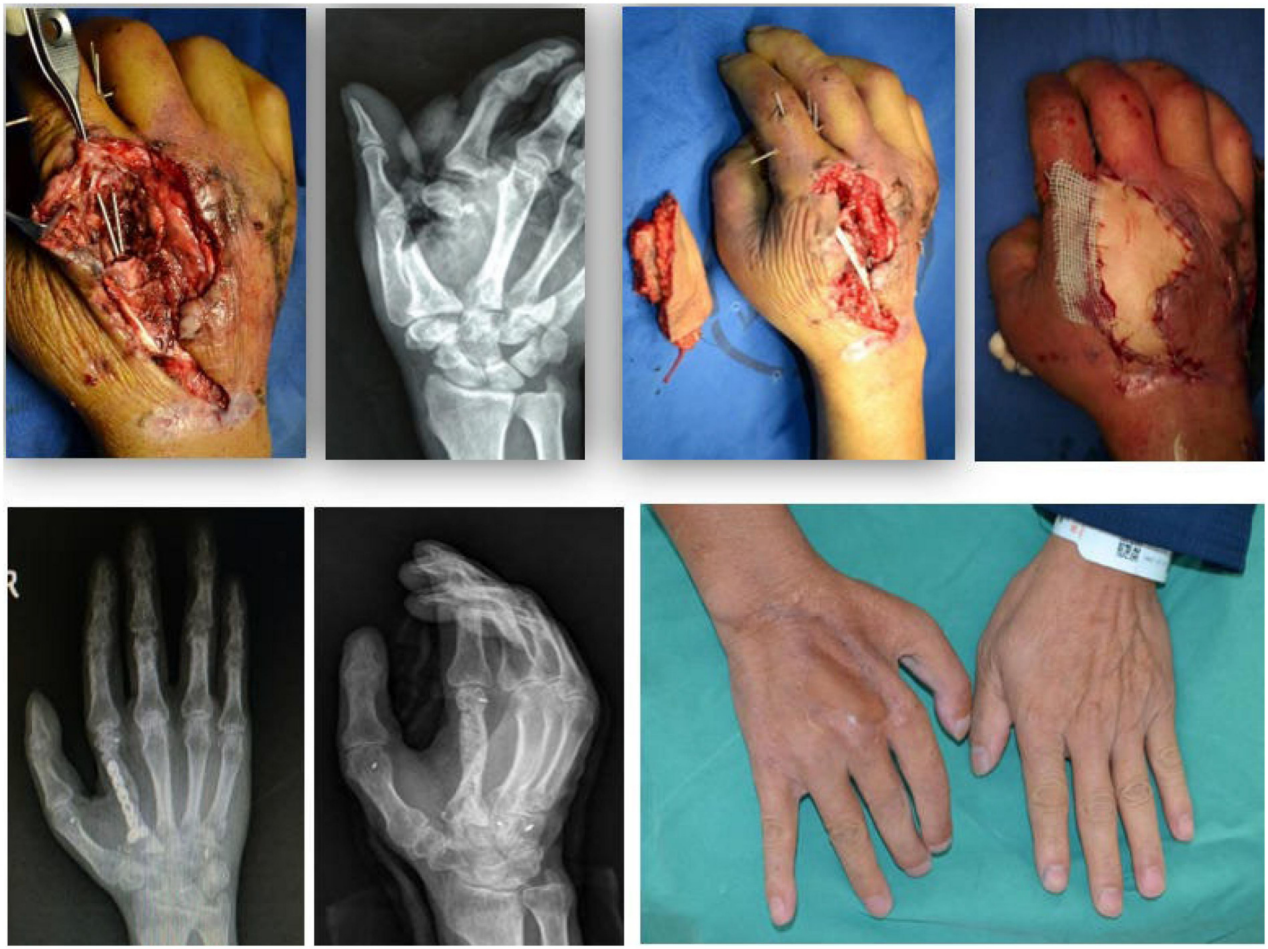


Postoperatively, the flap survived uneventfully. The iliac bone flap maintained an intact morphology, with no bone resorption observed. Clinical bone union of the bone graft was achieved at 3 months postoperatively, and the internal fixation device was removed at 8 months postoperatively. At the 36-month postoperative follow-up, the right hand function of the patient had returned to near-normal, which fully met the needs of daily living (Figure 2).

### 3.5 Case 2

A 32-year-old male patient was admitted to hospital due to right thumb avulsion injury caused by mechanical crush injury. Emergency debridement and revision of the right thumb stump were performed initially. On the 7th day after injury, the patient underwent right thumb reconstruction surgery.

Intraoperatively, a right hallux toenail flap and a second toe osseotendinous flap (sharing a common vascular pedicle) were harvested. The right thumb was reconstructed using a wrapping and combining technique with these two flaps. Defects at the donor foot (right foot) included: A 9 cm × 5 cm skin defect on the hallux; A 5 cm-long defect involving the phalanx and joint of the second toe.Concurrently, a right free perforator osteocutaneous flap of the superficial circumflex iliac artery (SCIA) was transplanted to reconstruct the complex defect of the donor foot. The cutaneous component of the SCIA flap measured 11 cm × 5.5 cm, and the bone flap dimensions were 4.0 cm × 1.0 cm × 1.0 cm. Additionally, a free iliac bone graft was harvested from the iliac region to repair the distal phalangeal defect of the hallux. Both the SCIA bone flap and the iliac bone graft were fixed with Kirschner wires.

**Figure 3:**
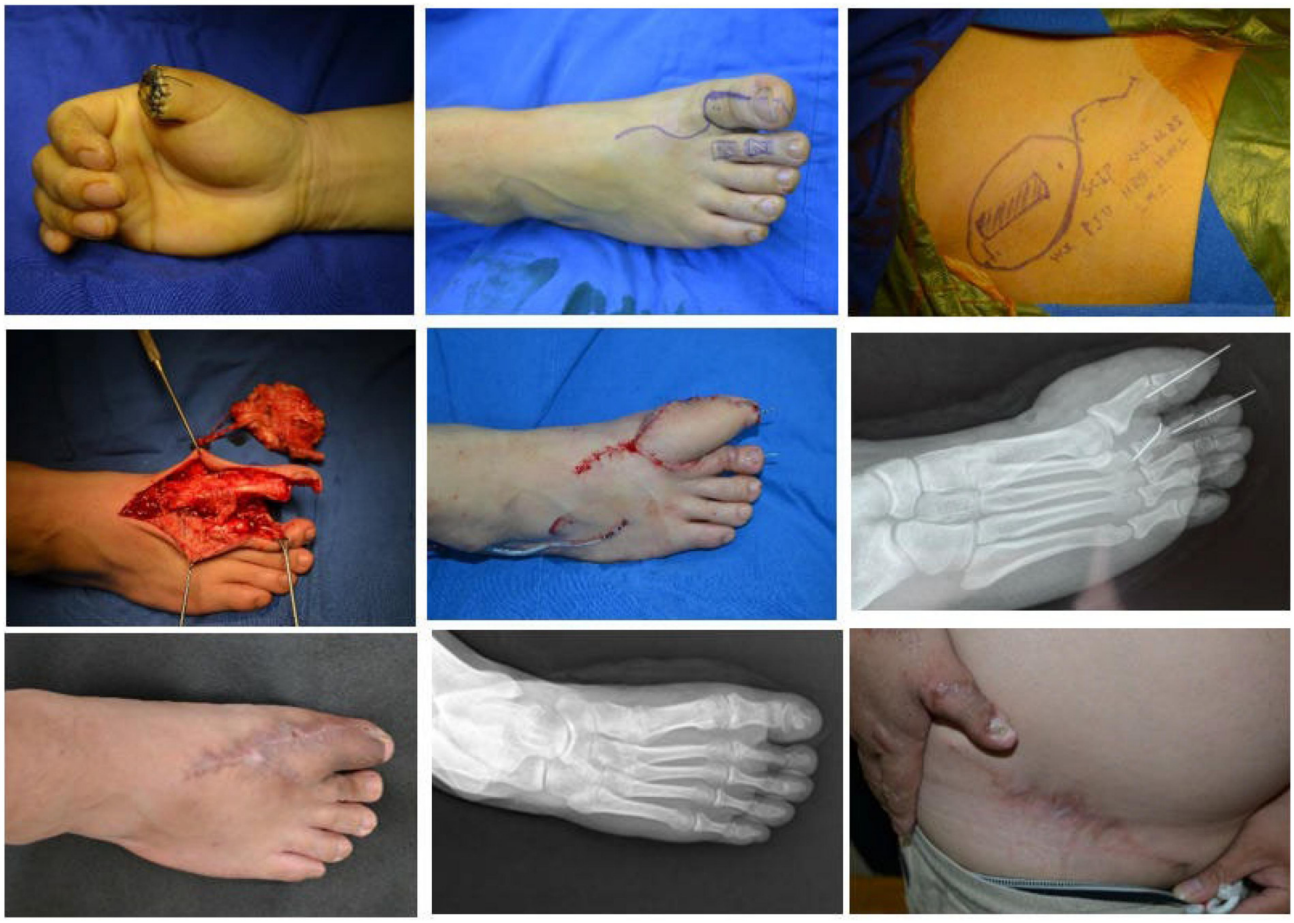


Postoperatively, the flap survived uneventfully. Three months later, flap revision surgery was performed due to local bulkiness of the reconstructed area. During postoperative follow-up:

1. The right foot had a satisfactory appearance, with no pain reported during daily shoe-wearing or physical activities;
2. The linear scar at the iliac donor site was inconspicuous;
3. X-ray examinations showed that the second toe bone flap maintained an intact morphology, achieved good bony union, and no bone resorption was observed (Figure 3).

## 4 Discussion

### 4.1 Treatment options for bone and soft tissue defects of the hand and foot

Clinically, a common approach for repairing bone and soft tissue defects of the hand and foot involves non-vascularized allogeneic or autologous bone grafting to address bone defects, followed by pedicled or free flap transplantation for skin defect coverage (4, 5). This method reliably reconstructs wounds with minimal bone defects; however, in cases of severe surrounding soft tissue injury, the nutritional supply to the bone ends adjacent to the defect is compromised. Consequently, simple bone grafts—relying on creeping substitution—often fail to achieve satisfactory union. Particularly in patients with osteoporosis or large bone defects, non-vascularized bone grafts are prone to complications such as bone resorption and nonunion.

Compared with simple bone grafting, induced membrane osteogenesis accelerates the creeping substitution of osteocytes within the graft by leveraging the abundant blood supply within the membrane. Nevertheless, this technique requires multiple surgical procedures and a 6–8 week period to form a stable induced membrane. This not only increases patient suffering and medical costs but also may cause patients to miss the optimal window for early hand/foot rehabilitation training, leading to varying degrees of hand or foot dysfunction.

In contrast to the aforementioned two methods, one-stage free osteocutaneous flap transplantation simultaneously achieves wound coverage and vascularized bone grafting when both bone and soft tissue defects coexist. The bone flap, with its inherent blood supply, exhibits robust viability. Unlike grafts dependent on creeping substitution, the healing process of the vascularized bone flap resembles that of a normal fracture, enabling patients to achieve faster and more effective bone structure reconstruction (6, 7).

The fibular osteocutaneous flap is the most commonly used vascularized bone flap in microsurgical bone transplantation (8). Despite its favorable clinical outcomes, several studies have reported donor site morbidity, including ankle pain, calf muscle contracture, common peroneal nerve injury, and insufficient foot perfusion due to sacrifice of the major calf blood vessels (9). Furthermore, for hand and foot reconstruction, the fibular flap is relatively large and lacks cancellous bone, making it difficult to perform subtle trimming of the bone graft. Thus, it is not suitable for reconstructing most complex hand and foot defects.

Conversely, donor sites such as the medial femoral condyle, radius, and lateral humeral condyle provide bone flaps rich in cancellous bone but with limited bone volume, restricting their application to small bone defects of the hand and foot. The iliac bone flap nourished by the deep circumflex iliac artery can be harvested with a large bone volume and can be combined with a cutaneous flap via perforating vessels, making it suitable for reconstructing wounds with large bone defects. However, its clinical application is limited by donor site morbidity and flap bulkiness.

Since 2011, the authors have designed the perforator osteocutaneous flap of the superficial circumflex iliac artery (SCIA) by utilizing the vessels of the deep branch of the SCIA that nourish the anterior superior iliac spine (ASIS)—building on the existing SCIA perforator flap. This modification not only significantly reduces flap thickness and donor site morbidity but also obtains an iliac bone flap with reliable blood supply. 4.2 Anatomical Basis of the SCIA Perforator Osteocutaneous Flap.

The superficial circumflex iliac artery (SCIA) originates from the femoral artery approximately 2.5 cm below the inguinal ligament, with a diameter of 1.0–1.6 mm at its origin. It divides into superficial and deep branches 1.5–3 cm lateral to the femoral artery.

The superficial branch pierces the deep fascia immediately after origination and courses toward the ASIS, giving off multiple small perforating vessels along its path to nourish the inguinal skin.

The deep branch travels superolaterally beneath the deep fascia, issuing muscular branches and musculocutaneous perforators. After piercing the deep fascia at the lateral margin of the sartorius muscle, it gives off cutaneous perforators to nourish the anterolateral iliac abdominal skin. Its terminal branch passes beneath the anterolateral femoral cutaneous nerve to the ASIS region, where it emits periosteal branches to nourish the anterolateral iliac crest.

Venous drainage is mediated by accompanying veins, as well as the superficial circumflex iliac vein (SCIV) and its branches, which run in the subcutaneous fat layer.Anatomical variations were observed in this study:In 2 cases (2/62), the superficial and deep branches originated separately from the femoral artery (no common trunk);In 12 cases (12/62), the superficial branch was relatively large, and the iliac bone flap was primarily nourished by this branch.

Based on the actual intraoperative anatomical findings, the SCIA perforator osteocutaneous flap can be harvested in multiple configurations (12, 13):

1. A chimeric osteocutaneous flap (superficial branch nourishing the cutaneous component, deep branch nourishing the bone component);
2. A perforator osteocutaneous flap pedicled on the deep branch of the SCIA;
3. A perforator osteocutaneous flap pedicled on the superficial branch of the SCIA.

### 4.2 Comparison with previous studies and study contributions

While the SCIA perforator osteocutaneous flap has been described in previous literature, our study differs from and contributes to existing knowledge in several key aspects.

Early reports, such as those by Iida et al. (10) and Yoshimatsu et al. (11), primarily explored the anatomical feasibility and initial clinical applications, often in head and neck reconstruction or small series of varied defects. In contrast, our study presents the largest clinical cohort to date (62 cases) specifically targeting composite defects of the extremities.

Furthermore, our findings expand the application of this flap to a specific clinical scenario: the reconstruction of donor-site lesions after digit reconstruction. We demonstrated that the SCIA osteocutaneous flap is uniquely suited for these cases because it provides high-quality cancellous bone that mimics the anatomical requirements of the foot or hand donor site.

Compared to the series reported by Yamashita et al. (12), which focused on extensive forefoot defects, our study emphasizes moderate-sized defects (2–8 cm of bone) where the preservation of major limb vessels (e.g., the peroneal or radial arteries) is paramount. By providing a detailed breakdown of complications and long-term functional recovery, this study offers more robust evidence for the reliability and safety of the SCIA osteocutaneous flap as a primary option for extremity reconstruction.

### 4.4 Advantages, disadvantages, and surgical considerations of the SCIA perforator osteocutaneous flap

#### 4.4.1 Advantages

1. The donor site can be primarily closed easily, with an inconspicuous scar;
2. Minimal donor site morbidity: most dissections are performed in the superficial layer of the abdominal wall, preserving the integrity of the abdominal wall muscles;
3. Large available flap area: the maximum flap area in this study was 13 cm × 5.5 cm, which meets the requirements for hand and foot wound reconstruction;
4. Thin flap thickness: the dissection plane lies between the superficial and deep fat layers;
5. Sufficient vascular pedicle length (up to approximately 9 cm in this study), meeting the requirements for free flap transplantation;
6. Flap sensation can be reconstructed by anastomosing the lateral cutaneous branch of the intercostal nerve within the flap (14).

#### 4.4.2 Disadvantages

1. Limited vascular supply to the iliac bone flap: insufficient blood supply may occur when harvesting a long or wide iliac bone flap. In this study, when an iliac bone flap of 8 cm × 2 cm × 1.5 cm was harvested, only minimal oozing was observed at the distal end of the bone osteotomy surface;
2. Small diameter of the pedicle vessels, resulting in poor matching with recipient site vessels;
3. Paresthesia in the anterolateral femoral cutaneous nerve distribution area, which is associated with intraoperative traction or injury to the nerve.

#### 4.4.3 Surgical considerations

1. The anterior osteotomy line of the iliac bone flap should be placed 2 cm posterior to the ASIS to preserve the integrity of the ASIS and avoid donor site morbidity;
2. The periosteal branches above the ASIS should be carefully dissected subperiosteally with a scalpel to prevent injury to these nutrient vessels. The blood supply to the iliac bone flap is provided by small vessels penetrating the periosteum; thus, the bone flap should be harvested without detaching the periosteum from the iliac crest;
3. Adequate hemostasis and drainage at the donor site are essential to prevent postoperative iliac hematoma and infection.

### 4.5 Limitations

This study has several limitations that should be noted. First, as a retrospective, single-center analysis, the study is subject to inherent selection biases and may lack the generalizability of multi-center trials. Second, the absence of a control group treated with alternative methods (such as the free fibular flap or conventional iliac bone flap) limits our ability to perform a direct comparative evaluation of therapeutic efficacy. Third, functional outcomes were primarily assessed qualitatively based on clinical examination and patient self-reports. Due to the long duration of the study period (spanning over a decade), standardized functional scoring systems (e.g., DASH or AOFAS scores) were not consistently available for all patients. Future prospective, multi-center studies with standardized functional metrics are needed to further validate these findings.

## 5 Conclusion

The SCIA perforator osteocutaneous flap represents a viable alternative for the reconstruction of complex defects, offering unique advantages in donor site morbidity while presenting its own set of technical challenges. While bone union was achieved in most cases, the occurrence of bone resorption (4.8%) and non-union (1.6%) highlights the need for careful patient selection and precise surgical execution.

## Data Availability

The original contributions presented in the study are included in the article/Supplementary Material, further inquiries can be directed to the corresponding authors.
